# Different Effects of Nicotine and N-Stearoyl-ethanolamine on Episodic Memory and Brain Mitochondria of α7 Nicotinic Acetylcholine Receptor Knockout Mice

**DOI:** 10.3390/biom10020226

**Published:** 2020-02-03

**Authors:** Olena Lykhmus, Olena Kalashnyk, Kateryna Uspenska, Tetyana Horid’ko, Halyna Kosyakova, Serhiy Komisarenko, Maryna Skok

**Affiliations:** Palladin Institute of Biochemistry, 01030 Kyiv, Ukraine; olenalykhmus@gmail.com (O.L.); o.kalashnyk56@gmail.com (O.K.); kate.uspenska@gmail.com (K.U.); tangori@ukr.net (T.H.); kosiakova@hotmail.com (H.K.); svk@biochem.kiev.ua (S.K.)

**Keywords:** nicotinic acetylcholine receptor, nicotine, N-stearoylethanolamine, α7 knockout mice, memory, mitochondria

## Abstract

Nicotinic acetylcholine receptors of α7 subtype (α7 nAChRs) are involved in regulating neuroinflammation and cognitive functions. Correspondingly, α7-/- mice demonstrate pro-inflammatory phenotype and impaired episodic memory. In addition, nAChRs expressed in mitochondria regulate the release of pro-apoptotic factors like cytochrome c. Here we studied whether the cognitive deficiency of α7-/- mice can be cured by oral consumption of either nicotine or N-stearoylethanolamine (NSE), a lipid possessing anti-inflammatory, cannabimimetic and membrane-stabilizing activity. Mice were examined in Novel Object Recognition behavioral test, their blood, brains and brain mitochondria were tested for the levels of interleukin-6, various nAChR subtypes and cytochrome c released by ELISA. The data presented demonstrate that both substances stimulated the raise of interleukin-6 in the blood and improved episodic memory of α7-/- mice. However, NSE improved, while nicotine worsened the brain mitochondria sustainability to apoptogenic stimuli, as shown by either decreased or increased amounts of cytochrome c released. Both nicotine and NSE up-regulated α4β2 nAChRs in the brain; NSE up-regulated, while nicotine down-regulated α9-containing nAChRs in the brain mitochondria. It is concluded that the level of alternative nAChR subtypes in the brain is critically important for memory and mitochondria sustainability in the absence of α7 nAChRs.

## 1. Introduction

Nicotinic acetylcholine receptors (nAChRs) are ligand-gated ion channels mediating fast synaptic transmission in muscles and autonomic ganglia, regulating the neurotransmitter release in the brain and involved in regulating the survival, proliferation and adhesion of many non-excitable cells [1,2,3,4]. The nAChRs of α7 subtype are involved in cholinergic anti-inflammatory pathway by attenuating the production of pro-inflammatory cytokines IL-1β and TNFα [5]. Correspondingly, the decrease of α7 nAChRs in the brain was observed upon neuroinflammation induced by regular injections of bacterial lipopolysaccharide (LPS) and, *vice versa*, decreasing the level of α7 nAChRs by immunization with α7 extracellular domain (1-208) resulted in neuroinflammation [6]. Neuroinflammation is closely related to cognition: the memory decline observed upon neurodegenerative disorders like Alzheimer’s disease is accompanied or even preceded by neuroinflammation [7,8,9].

In addition to the cell plasma membrane, several nAChR subtypes, including α7-containing subtype, were found in the outer membrane of mitochondria, where they regulate the release of pro-apoptotic factors like cytochrome c (cyt c) caused by the increase of intracellular Ca^2+^ or reactive oxygen species [10]. The decrease of mitochondrial nAChRs observed upon inflammation was shown to be accompanied by increased cyt c release under the effect of Ca^2+^ or H_2_O_2_ [11].

Mutant mice lacking α7 nAChRs (α7-/-) were shown to possess pro-inflammatory phenotype with increased level of pro-inflammatory cytokines in the blood, spleen and brain [12,13,14]. Initial studies did not demonstrate any cognitive differences between the wild-type (WT) and α7-/- mice [15]. Later it was found that α7-/- mice were different in the anxiety-related behavior [16], attention [17,18] and visual activity [19]. In our hands, the α7-/- mice demonstrated higher locomotor and explorative activity but impaired episodic memory measured in the Novel Object Recognition (NOR) test [20]. Their mitochondria were more sensitive to Ca^2+^ compared to mitochondria of the WT mice. However, the difference was not dramatic due to compensatory up-regulation of mitochondrial α4- and α9-containing nAChRs [21].

Previously we reported that episodic memory decline and mitochondrial impairment in LPS-treated mice can be prevented by treatment with N-stearoylethanolamine (NSE), a natural component of cellular membranes possessing cannabimimetic, anti-inflammatory and membrane-stabilizing activity [22]. Here we asked if NSE can improve memory and stabilize mitochondria in the absence of α7 nAChRs in α7-/- mice. The effect of NSE was compared with that of nicotine known to activate α4β2 nAChRs critically important for memory and cognition [3,23].

## 2. Materials and Methods

### 2.1. Reagents

All reagents were of chemical grade and purchased from Sigma-Aldrich (Saint Louis, MO, USA), unless specially indicated. Antibodies against α3(181-192), α4(181-192), α5(180-191), α7(179-190), α9(11-23) or α7(1-208)nAChR fragments and rabbit cyt *c*-specific antibodies were generated using methods previously developed in our lab [24,25,26,27,28]. The antibodies were biotinylated according to standard procedures [29]. Mouse IL-6 ELISA Ready-SET (Ref # 88-7064-88) was purchased from ALT Ukraine Ltd. (Kyiv, Ukraine, official representative of Thermo Fisher Scientific in Ukraine). NSE was synthesized in the Department of Lipid Biochemistry at Palladin Institute of Biochemistry as described previously [22,30]. 

### 2.2. Animals

We used the C57Bl/6 (WT) and α7-/- male mice 3-5 months of age. The colony of α7-/- mice was developed in Kyiv from a nucleus kindly presented by Dr. Uwe Maskos from Institut Pasteur, Paris. Mice were kept in the animal facility of Palladin Institute of Biochemistry NAS of Ukraine, were housed in quiet, temperature-controlled rooms and provided with water and food pellets *ad libitum*. Before removing the brains, mice were sacrificed by cervical dislocation. All procedures complied with the ARRIVE guidelines, were carried out in accordance with the Directive 2010/63/EU for animal experiments and were approved by the Animal Care and Use Committee of Palladin Institute of Biochemistry.

### 2.3. Experimental Schedule and the Brain Preparations

The α7-/- mice were randomly divided into three groups, five animals in each. One group was fed with NSE *per os*, 50 mg kg^−1^, 50 µL per mouse during 7 days. Another group obtained nicotine with the drinking water (200 µL/L) during three weeks. The blood was taken from the tail vein on day 0 (all groups), day 14 (one week after cessation of NSE treatment) or day 21 (nicotine). The mice were examined in behavioral test on days 0, 7 and 14 or 21 and sacrificed. 

The WT mice were divided into two groups, three animals in each. Both groups were injected intraperitoneally with lipopolysaccharide (LPS, *E. coli* strain 055:B5, 1.5 mg/kg) in PBS. One group of mice obtained four subcutaneous injections of recombinant IL-6 (750 pg in 50 µL) once per day starting from day 4 after LPS injection. The mice were examined in behavioral test on days 0 (before LPS injection), 7 and 14 and sacrificed.

The mouse brains were homogenized in a glass homogenizer in sucrose-containing buffer (10 mM HEPES, 1 mM EGTA, 200 mM sucrose) and centrifuged (1500× *g*, 10 min, 4 °C). The pellet was considered as a non-mitochondria fraction, while the supernatant was additionally centrifuged (2× 10 min, 8500× *g* at 4 °C) and the pellet (mitochondria) was resuspended in mitochondria incubation medium and used for functional cyt *c* release assay (see Section 2.5). The pellets of both mitochondria (collected after cyt c release) and non-mitochondria fractions were used to prepare the detergent lysates, as described previously [6]. The purity of mitochondrial and non-mitochondrial brain fractions obtained was evaluated by ELISA using the antibodies against different cellular compartments, as described previously [31].

### 2.4. ELISAs

The level of various nAChR subunits within the brain or mitochondria preparations was studied as described previously [6]. Briefly, the immunoplates (Nunc MaxiSorp, purchased from ALT Ukraine Ltd. (Kyiv, Ukraine, official representative of Thermo Fisher Scientific in Ukraine). were coated with rabbit α7(1-208)-specific antibody (20 µg/mL), blocked with 1% BSA, and the detergent lysates of brain tissue or mitochondria were applied into the wells (1 µg of protein per 0.05 mL per well) for 2h at 37 °C. The plates were washed with water and the second biotinylated α3(181-192)-, α4(181-192)-, α7(179-190)-, α9(11-23)-, β2(190-200)- or β4(190-200)-specific antibody was applied for additional 2 h being revealed with neutravidin-peroxidase conjugate and *o*-phenylendiamine-containing substrate solution. The level of IL-6 in the blood sera was measured using IL-6-specific ELISA kit and procedure recommended by manufacturer. The optical density of ELISA plates was read using Stat-Fax 2000 ELISA Reader (Awareness Technologies, Westport, CT, USA). 

### 2.5. Cyt c Release Assay

The purified brain mitochondria (120 µg of protein per ml) were incubated with either 0.9 µM CaCl_2_ or 0.5 mM H_2_O_2_ for 5 min at room temperature and were immediately pelleted by centrifugation (10 min, 7000× *g*) at 4 °C. The incubation medium contained 10 mM HEPES, 125 mM KCl, 25 mM NaCl, 5 mM sodium succinate and 0.1 mM Pi(K), pH 7.4. The mitochondria supernatants were collected and tested for the presence of cyt *c* by sandwich assay as described previously [10,28].

### 2.6. Behavioral Test

Mice were tested in the “Novel Object Recognition” (NOR) behavioral test [6,32] prior and each week post-treatments. Briefly, the animals were individually placed in a rectangular novel open field containing two identical objects with distinctive features (shape and texture). The animals were subjected to a 10-min session of exploration of the objects followed by 10 min in a waiting cage. During the second 10-min session, one of the objects was replaced by a novel one and we scored the time spent in contact with each object. It is widely acknowledged that rodents spontaneously explore novel objects by touching the objects with their nose and prefer novel objects to familiar ones that reflect their episodic memory [33,34]. Therefore, the time spent in contact with each object reflects the time of exploration for the object. The results of NOR test are presented as Discrimination Index (DI) calculated as the difference in the number of “novel” and “famous” object explorations divided by the total number of explorations of two identical objects. 

### 2.7. Statistical Analysis

ELISA experiments have been performed in triplicates and mean values for individual mice were used for statistical analysis using one-way ANOVA test. Behavioral tests were performed three times (days 0, 7 and 14 or 21) for each mouse and counts of individual mice were taken for statistical analysis. The data are presented as Mean ± SD; * *p* < 0.05; ** *p* < 0.005; *** *p* < 0.0005.

## 3. Results

**Figure 1:**
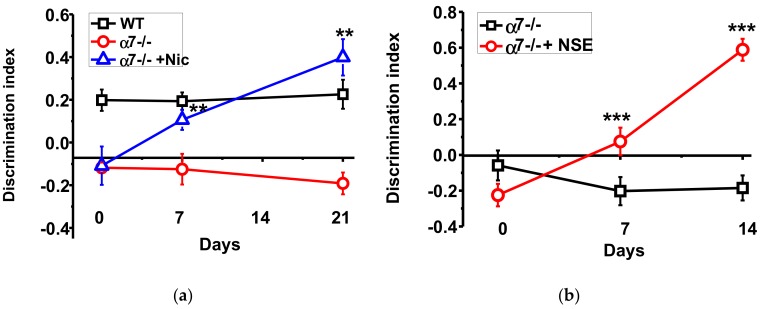
Memory (discrimination index studied in NOR test) of α7-/- mice under the effect of either nicotine (**a**) or NSE (**b**). Each point on the curve corresponds to M ± SD, n-5. ** *p* < 0.005; *** *p* < 0.0005 compared to Day 0 (non-treated mice). WT—wild-type C57Bl mice.

The α7-/- mice demonstrated significantly worse episodic memory compared to the WT mice; this difference was maintained during all experimental period. Consuming nicotine with the drinking water increased the discrimination index of the new *vs* familiar objects recognition already after seven days. After three weeks of nicotine consumption the memory of α7-/- mice was similar, or even better, than that of the WT mice (Figure 1a). NSE, given during a week, improved memory of the α7-/- mice and the effect was further developed a week afterwards (Figure 1b).

The brains of mice were fractionated into mitochondria and non-mitochondria fractions. According to ELISA with the cell compartment-specific antibodies, the mitochondria fraction was positive for mitochondria-specific voltage-dependent anion channels (VDAC) and negative for nuclear-specific lamin B1 and endoplasmic reticulum-specific IRE-1α, while the “non-mitochondria” fraction contained lamin 1B and IRE-1α, but not VDAC [31].

**Figure 2:**
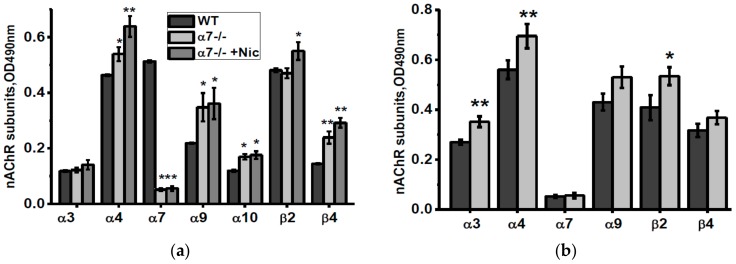
nAChR subunits composition in the brain (w/o mitochondria) fractions of α7-/- mice under the effect of either nicotine (**a**) or NSE (**b**). Each column corresponds to M ± SD, n-5. * *p* < 0.05; ** *p* < 0.005 compared to non-treated WT mice (**a**) or α7-/- mice (**b**).

The non-mitochondria brain fraction of α7-/- mice contained elevated amounts of α4, α9, α10 and β4 nAChR subunits compared to corresponding fraction of the WT mice (Figure 2a). Consuming nicotine did not influence α9 and α10 subunits but increased α4, β2 and, less, β4 subunits. Consuming NSE also increased α4, β2 and, in addition, α3 nAChR subunits (Figure 2b).

Interleukin-6 (IL-6) is usually regarded as one of pro-inflammatory cytokines. However, in addition, it has been regarded as a neurotrophic factor [35]. Previously we reported that subcutaneous injections of recombinant IL-6 up-regulated α3β4 and α4β2 nAChRs in the brain of α7-/- mice and improved their episodic memory [20]. Here we asked if IL-6 can prevent pathogenic effect of inflammation in wild-type C57Bl/6 mice.

**Figure 3:**
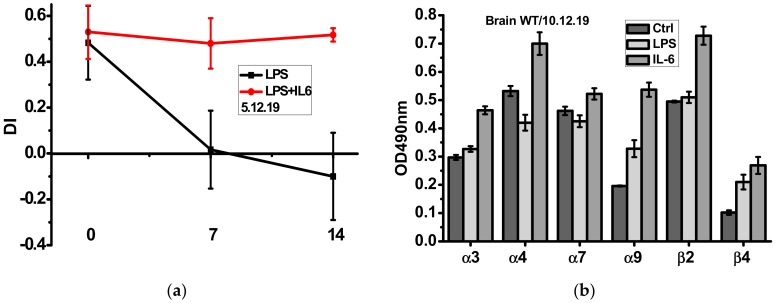
Episodic memory (discrimination index studied in NOR test) (**a**) and nAChR subunit composition in the brain (w/o mitochondria, (**b**) of the wild-type mice injected with either LPS alone or LPS and IL-6. Ctrl–non-injected mice. ** *p* < 0.005; *** *p* < 0.0005 compared to LPS-treated mice, *n* = 3.

As shown in Figure 3a, episodic memory of mice injected with LPS was gradually decreased during two weeks and this was not the case if mice were injected with recombinant IL-6 the first four days after LPS injection. Sandwich ELISA using the antibodies against different nAChR subunits demonstrated that IL-6 injections up-regulated α3, α4, α7, α9 and β2 nAChR subunits, some of them being down-regulated by LPS (Figure 3b).

**Figure 4:**
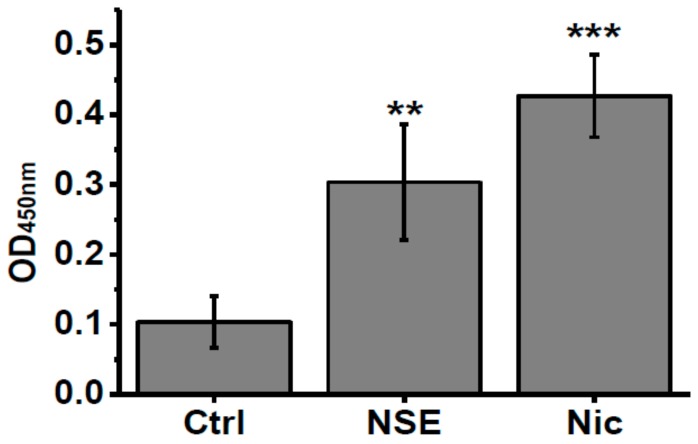
Level of IL-6 in the blood sera of α7-/- mice under the effect of either nicotine or NSE. Each column corresponds to M ± SD, n-5. ** *p* < 0.005; *** *p* < 0.0005 compared to non-treated α7-/- mice (Ctrl).

To check if nicotine or NSE influence the IL-6 level in α7-/- mice we measured this cytokine in the blood of mice under experiment. As shown in Figure 4, both nicotine and NSE significantly increased the amount of IL-6 in the blood of α7-/- mice at the time point when memory improvement was observed. Therefore, the memory-improving effects of both NSE and nicotine could be related to increased IL-6 levels.

**Figure 5:**
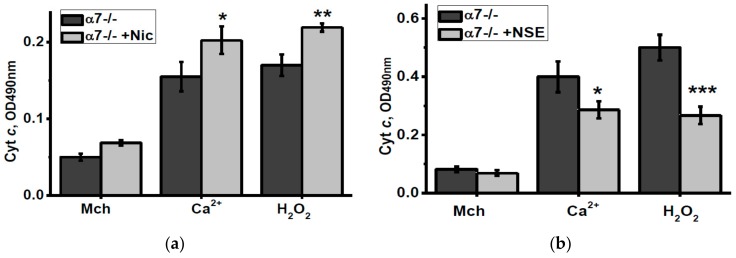
Level of cytochrome c (Cyt c) released from the brain mitochondria of α7-/- mice treated by either nicotine (**a**) or NSE (**b**) under the effect of 0.9 µM Ca^2+^ or 0.5 mM H_2_O_2_. Each column corresponds to M ± SD, n-5. * *p* < 0.05; ** *p* < 0.005; *** *p* < 0.0005 compared to non-treated α7-/- mice. Mch—cyt c released from non-treated mitochondria.

To check how nicotine or NSE consumption affected the brain mitochondria sustainability to apoptogenic influence, we measured the amounts of cyt c released from isolated mitochondria under the effect of either Ca^2+^ or H_2_O_2_. As shown in Figure 5, nicotine consumption increased, while NSE consumption attenuated cyt c release from mitochondria of α7-/- mice stimulated by these apoptogenic agents.

**Figure 6:**
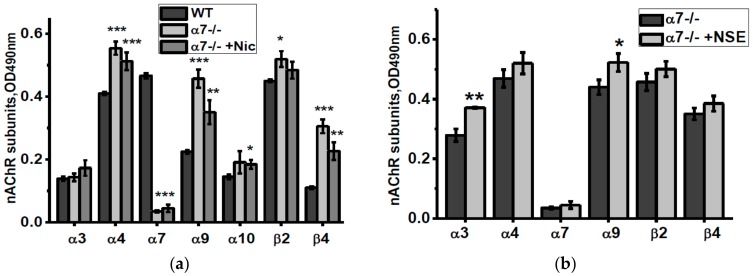
nAChR subunits composition in the brain mitochondria of α7-/- mice under the effect of either nicotine (**a**) or NSE (**b**). Each column corresponds to M ± SD, n-5. * *p* < 0.05; ** *p* < 0.005; *** *p* < 0.0005 compared to non-treated WT mice (**a**) or α7-/- mice (**b**).

According to ELISA data, mitochondria of α7-/- mice contained more α4, α9, β2 and β4 nAChR subunits compared to mitochondria of the WT mice (Figure 6a). Nicotine consumption decreased α9 and β4 nAChR subunits, while NSE consumption increased α3 and α9 subunits compared to mitochondria of non-treated α7-/- mice (Figure 6a,b).

## 4. Discussion

Our previous studies clearly showed that neuroinflammation is accompanied by the down-regulation of α7 nAChRs in the brain and, vice versa, decreasing the level of α7 nAChRs by corresponding antibodies results in neuroinflammation and memory deficiency [6]. In addition, we were the first to show the presence of nAChRs in the outer membrane of mitochondria and their involvement in the early stages of mitochondria-driven apoptosis [10]. Correspondingly, neuroinflammation negatively affected mitochondria sustainability [11]. These studies pushed us to search the approaches to overcome the pathological effects of neuroinflammation and α7 nAChR deficiency. In particular, it was shown that neuroinflammation can be prevented or even cured by activating α7 nAChRs with orthosteric agonist [36] or by up-regulating alternative nAChR subtypes with the growth factors produced by mesenchymal stem cells [37]. One of our previous papers described the efficiency of NSE to prevent neuroinflammation in the wild-type mice either treated with LPS of immunized with α7 nAChR extracellular domain 1-208 [22]. The present manuscript continues this set of studies by employing the “end-point” model of α7 nAChR deficiency—knockout mice completely lacking this nAChR subtype—to show that although up-regulation/activation of alternative nAChR subtypes is an efficient approach to support/improve cognitive functions, the most obvious way, like nicotine consumption, is not good because it negatively affects the brain mitochondria.

The data presented here demonstrate that episodic memory of α7-/- mice can be improved by either nicotine or NSE. However, NSE improved, while nicotine worsened the brain mitochondria sustainability to apoptogenic stimuli, as shown by either decreased or increased amounts of cyt c released from isolated mitochondria. These phenomena seem to correlate with the nAChR content/composition: both nicotine and NSE up-regulated α4β2 nAChRs in the brain; NSE up-regulated, while nicotine down-regulated α9-containing nAChRs in the brain mitochondria of α7-/- mice.

Both the mitochondria and non-mitochondria fractions of α7-/- mice contained elevated amounts of other nAChR subunits compared to corresponding fractions of the WT mice: α4, α9, β2 and β4 in mitochondria and α4, α9, α10 and β4 subunits in non-mitochondria. This data is in accord with our previous results [21] and is consistent with the idea that the absence of α7-containing nAChRs is compensated by up-regulation of alternative nAChR subtypes. According to established nAChR subunit combinations, these could be α4β2/α4β4 and α9α10nAChRs. In addition, we previously showed the presence of α9β4 combination in mitochondria of α7-/- mice and in rats subjected to partial hepatectomy by sandwich ELISA using a pair of α9- and β4-specific antibodies [21] that is also supported by the present data. The absence of α7-containing nAChRs did not significantly influence cyt c release from their mitochondria under the effect of either Ca^2+^ or H_2_O_2_ [21]; therefore, the nAChR subtypes expressed in compensation were sufficient to support mitochondria protection against apoptogenic stimuli. In contrast, the nAChRs up-regulated in non-mitochondria fraction of the brain (α4β4 and α9α10), potentially including plasma membrane nAChRs, were not sufficient to control inflammation [12] or to fully support episodic memory in α7-/- mice.

Both nicotine and NSE treatment up-regulated α4β2 nAChRs in non-mitochondria brain fraction and improved memory. This is consistent with the principal role of α4β2 nAChRs in learning and memory [23]. Interestingly, such improvement was accompanied by increased peripheral IL-6 production. Previously we found that recombinant IL-6 up-regulated α4β2 nAChRs in the brain of α7-/- mice and improved their memory [20]. Here we provide evidence that injections of recombinant IL-6 prevented episodic memory decline under the effect of LPS and up-regulated several nAChR subtypes in the brain of the wild-type mice. Therefore, it seems realistic to suggest that the effects of both nicotine and NSE in the brains of α7-/- mice are, at least partly, mediated by IL-6. In addition to its pro-inflammatory function, IL-6 is considered as a neurotrophic factor [35] and its effect appears to be sufficient to compensate the α7 nAChR deficiency, at least, related to episodic memory.

Interestingly, the effects of NSE or nicotine on the brain mitochondria were opposite: NSE increased mitochondria sustainability to apoptogenic influence and up-regulated α9-containing nAChRs, while nicotine decreased sustainability and down-regulated α9-containing nAChRs. Previously we reported that NSE stabilized mitochondria of LPS-treated WT mice and restored their α7 nAChRs decreased by inflammation [22] that is consistent with the present data. We also found that nicotine consumption by the WT mice facilitated the α4β2 nAChR trafficking to the liver mitochondria, did not influence other nAChR subtypes and did not affect the mitochondria sensitivity to Ca^2+^ [31]. Here we show that nicotine decreased the level of α9- and β4-containing nAChRs in the brain mitochondria of α7-/- mice (up-regulated to compensate the absence of α7 nAChRs) that worsened the mitochondria sustainability. This data indicates that the effect of nicotine on mitochondria is different in either the presence (WT) or absence of α7 nAChRs (α7-/-). Possibly, this could be explained by different consequences of nicotine interaction with either α7- or α9-containing nAChRs. Nicotine is an agonist for α7 nAChRs but is antagonistic towards α9 nAChRs [38]. In addition, different types of intracellular signaling were suggested for these two nAChR subtypes [39,40]. In one of our previous papers we showed the presence of α9-containing nAChRs in the brain of WT C57Bl/6 mice and their up-regulation in α7-/- mice [41]. Those data were in contradiction to previously established absence of α9 expression in the brain [42] and provoked certain criticism [43]. The results presented here support our previous findings and demonstrate that, at least in the brain mitochondria, the α9-containing nAChRs can be responsible for the sustainability to apoptogenic stimuli and for unusual effect of nicotine in α7-/- mice.

Taken together, the data presented here confirm the previously established important role of α7 nAChRs for cognitive processes like episodic memory [44,45] and suggest the approaches to improve it in the absence of this nAChR subtype. It is quite clear that memory impairment caused by α7 nAChR deficiency can be compensated by up-regulation of α4β2 nAChRs, which can be achieved by interventions stimulating neurotrophic factors (eg. IL-6) production. However, nicotine, which is a classical nAChR ligand stimulating α4β2 nAChRs, cannot be recommended for this purpose because it negatively affects the brain mitochondria. In addition to its previously established negative effect on mitochondria respiratory chain, that is receptor-independent [46], it down-regulates α9β4 nAChRs, which compensate the α7 nAChR deficiency, and makes mitochondria more sensitive to apoptogenic influence. In contrast, NSE positively affects both memory and mitochondria and, therefore, can be a drug of choice to restore the cognitive functions impaired by α7 nAChR deficiency. Although complete absence of α7 nAChRs is not described in the wild-type animals or humans, its partial deficiency may be critical, for example, upon inflammation.

NSE is a natural component of mammalian cell membranes, along with N-palmitoyl-ethanolamine, N-oleylethanolamine and N-linoleylethanolamine, which are important and physiologically relevant mediators of cell protection against various pathological conditions [47]. In addition to direct incorporation into the cell membranes, N-acylethanolamines were shown to interact with cannabinoid, vanyloid and nuclear PPAR receptors [48,49,50]. NSE was shown to possess cannabimimetic activity; however, its binding sites found in mouse brain are different from cannabinoid receptors [51]. Previous experiments demonstrated neuroprotective effect of NSE in the model of chronic morphine dependence [52,53] and its anti-acetylcholine esterase and pro-cognitive activity in the model of cholinergic deficiency caused by scopolamine [54]. We also showed that NSE restored memory and α7 nAChR content and stabilized mitochondria in the brains of WT mice either injected with LPS or immunized with the α7 extracellular domain (1-208) [22]. The present study confirms this data and shows the efficiency of NSE even in complete absence of α7 nAChRs. Production of endogenous N-acylethanolamines was shown to be dependent on α7 nAChR activation [55]; therefore, consumption of exogenous NSE by α7-/- mice can compensate the deficiency of endogenous compound. Correspondingly, one may suggest that N-acylethanolamines are mediators of at least a part of α7 nAChR physiological effects.

## 5. Conclusions

(1) In the absence of α7 nAChRs, the levels of α4β2 nAChRs in the brain and of α9 nAChRs in the brain mitochondria are critically important for memory and mitochondria sustainability, respectively.
(2) Both nicotine and NSE stimulate IL-6 production, which favor up-regulation of alternative nAChR subtypes in α7-/- and LPS-injected wild-type mice.
(3) Nicotine improves memory of α7-/- mice but negatively affects the brain mitochondria.
(4) NSE positively affects both memory and mitochondria and, therefore, can be a drug of choice to restore the cognitive functions impaired by α7 nAChR deficiency.
(5) Taking into account the established role of α7 nAChRs in neuroinflammation, the results of our study demonstrate a therapeutic potential of NSE in treating neuroinflammation-dependent neurodegenerative disorders, like Alzheimer disease.
